# CCBE1 in Cardiac Development and Disease

**DOI:** 10.3389/fgene.2022.836694

**Published:** 2022-02-09

**Authors:** Fernando Bonet, José M. Inácio, Oriol Bover, Sabrina B. Añez, José A. Belo

**Affiliations:** ^1^ Stem Cells and Development Laboratory, CEDOC, Chronic Diseases Research Centre, NOVA Medical School, Universidade Nova de Lisboa, Lisboa, Portugal; ^2^ Medicine Department, School of Medicine, University of Cádiz (UCA), Cádiz, Spain; ^3^ Research Unit, Biomedical Research and Innovation Institute of Cadiz (INiBICA), Puerta del Mar University Hospital, Cádiz, Spain

**Keywords:** cardiogenesis, cvd, CCBE1, Hennekam syndrome, lymphangiogenesis, proliferation, coronary vessels

## Abstract

The collagen- and calcium-binding EGF-like domains 1 (CCBE1) is a secreted protein extensively described as indispensable for lymphangiogenesis during development enhancing VEGF-C signaling. In human patients, mutations in *CCBE1* have been found to cause Hennekam syndrome, an inherited disease characterized by malformation of the lymphatic system that presents a wide variety of symptoms such as primary lymphedema, lymphangiectasia, and heart defects. Importantly, over the last decade, an essential role for CCBE1 during heart development is being uncovered. In mice, *Ccbe1* expression was initially detected in distinct cardiac progenitors such as first and second heart field, and the proepicardium. More recently, *Ccbe1* expression was identified in the epicardium and sinus venosus (SV) myocardium at E11.5–E13.5, the stage when SV endocardium–derived (VEGF-C dependent) coronary vessels start to form. Concordantly, CCBE1 is required for the correct formation of the coronary vessels and the coronary artery stem in the mouse. Additionally, *Ccbe1* was found to be enriched in mouse embryonic stem cells (ESC) and revealed as a new essential gene for the differentiation of ESC-derived early cardiac precursor cell lineages. Here, we bring an up-to-date review on the role of CCBE1 in cardiac development, function, and human disease implications. Finally, we envisage the potential of this molecule’s functions from a regenerative medicine perspective, particularly novel therapeutic strategies for heart disease.

## Introduction

Cardiovascular diseases, namely coronary artery disease (CAD) and inherited heart defects, are the most prevalent cause of lethality among human patients worldwide. It is also the most frequent type of genetic-caused disability in human patients (Liu et al., 2019). Heart development is a complex process that is highly regulated, leading to the formation of a four-chambered heart. The primitive heart tube is formed from the cardiac mesoderm of the cardiac crescent named First Heart Field (FHF) (Zaffran et al., 2004). The heart tube grows at both ends by addition of a subset of cardiogenic cells from the cardiac crescent, dubbed second heart field (SHF), into its anterior (arterial) and posterior (venous) poles (Mjaatvedt et al., 2001; Waldo et al., 2001; Moorman and Christoffels, 2003; Buckingham et al., 2005; Kelly 2012). The linear heart tube undergoes a rightward looping, and the outer layer of the myocardium starts proliferating, contributing to the formation of the future cardiac chambers (Moorman et al., 2003). Afterwards, the septated four-chambered fetal heart is generated including an extracardiac cell source named cardiac neural crest (CNC; Le Lievre and Le Douarin, 1975; Waldo et al., 1998; Jain et al., 2011). A second extracardiac cell source, the proepicardium (PE), will cover the myocardium forming the epicardial layer that will give rise to distinct cardiovascular embryonic cell linages, such as endothelial and smooth muscle cells of the coronary vessels, atrioventricular cushion mesenchymal cells, and cardiac fibroblasts (Poelmann et al., 1993; Mikawa and Gourdie, 1996; Dettman et al., 1998; Männer et al., 2001; Hirose et al., 2006; Katz et al., 2012; Cano et al., 2016).

Early in cardiogenesis, the heart consists of two concentric layers, a thin outer myocardium and the inner endocardium. At these stages, oxygen is easily supplied to the myocardium by passive diffusion from blood flowing through its lumen. However, myocardial growth triggers an invasion of endothelial cells (ECs) that undergo vasculogenesis to form an immature coronary vascular plexus (Zeini et al., 2009; Red-Horse et al., 2010). Then, this coronary plexus expands and branches, via sprouting angiogenesis, covering and infiltrating the entire myocardium. Coronary endothelium arise from a variety of sources including the sinus venosus (SV) endocardium (Red-Horse et al., 2010; Chen et al., 2014a), the ventricular endocardium (Wu et al., 2012), and the PE (Katz et al., 2012; Cano et al., 2016). The primitive coronary vascular network eventually anastomoses with the aorta allowing blood supply to the myocardium (Waldo et al., 1990). Subsequently, the coronary plexus undergoes a remodeling stage giving rise to a hierarchal vasculature tree composed of arteries, veins and capillaries (Waldo et al., 1990; Vrancken Peeters et al., 1997; Tomanek 2005). The result is a mature coronary vasculature system that efficiently supports the oxygenation of myocardial tissue. CAD is the leading cause of death worldwide and is characterized by a decrease or blockage of the flow of oxygenated blood to the heart muscle (Virani et al., 2021). Understanding the cellular and molecular mechanisms driving heart formation (myocardium and coronary vasculature development), may offer novel approaches for repairing and regenerating heart diseases.

The collagen- and calcium-binding EGF-like domains 1 (CCBE1) is an extracellular matrix (ECM) protein best known for its essential role in lymphatic vasculature development (Hogan et al., 2009; Bos et al., 2011). Mutations in *CCBE1* were identified in human patients with Hennekam syndrome (HS), a rare autosomal recessive disorder of lymphatic development that presents a wide variety of symptoms including primary lymphedema, and heart defects (Van Balkom et al., 2002; Alders et al., 2009; Connell et al., 2010; Connell et al., 2012; Alders et al., 2013; Shah et al., 2013). Most recently, CCBE1 has been reported as an important protein in cardiovascular development (Furtado et al., 2014; Bonet et al., 2018; Bover et al., 2018). In this review, we focus on the current knowledge of the role of CCBE1 in the context of heart development and the future perspectives regarding its implications for translational/regenerative medicine. First, we explain its structure, mechanisms of action and the distinct phenotypes associated to CCBE1 mutations in distinct animal models. Second, we describe *Ccbe1* expression during heart development and its function during both early cardiogenesis and coronary vasculature development. Finally, we inquire in the transcriptional regulation of CCBE1 in distinct contexts. Moreover, we discuss how the knowledge gained on the role of CCBE1 during cardiogenesis can be used to generate new therapeutic approaches for the treatment of congenital heart disease and for improving cardiac function in situations of ischemic heart disease.

## CCBE1: Structure and Function

*CCBE1* was first identified in a genetic study over 18q21-qter chromosomal region in the breast and prostate cancer cell lines aiming to identify genes whose expression was downregulated (Yamamoto and Yamamoto, 2007). Later, *CCBE1* was detected in a differential screening designated to identify genes enriched in heart progenitor cells unveiling its potential role in different biological processes (Bento et al., 2011).

**FIGURE 1:**
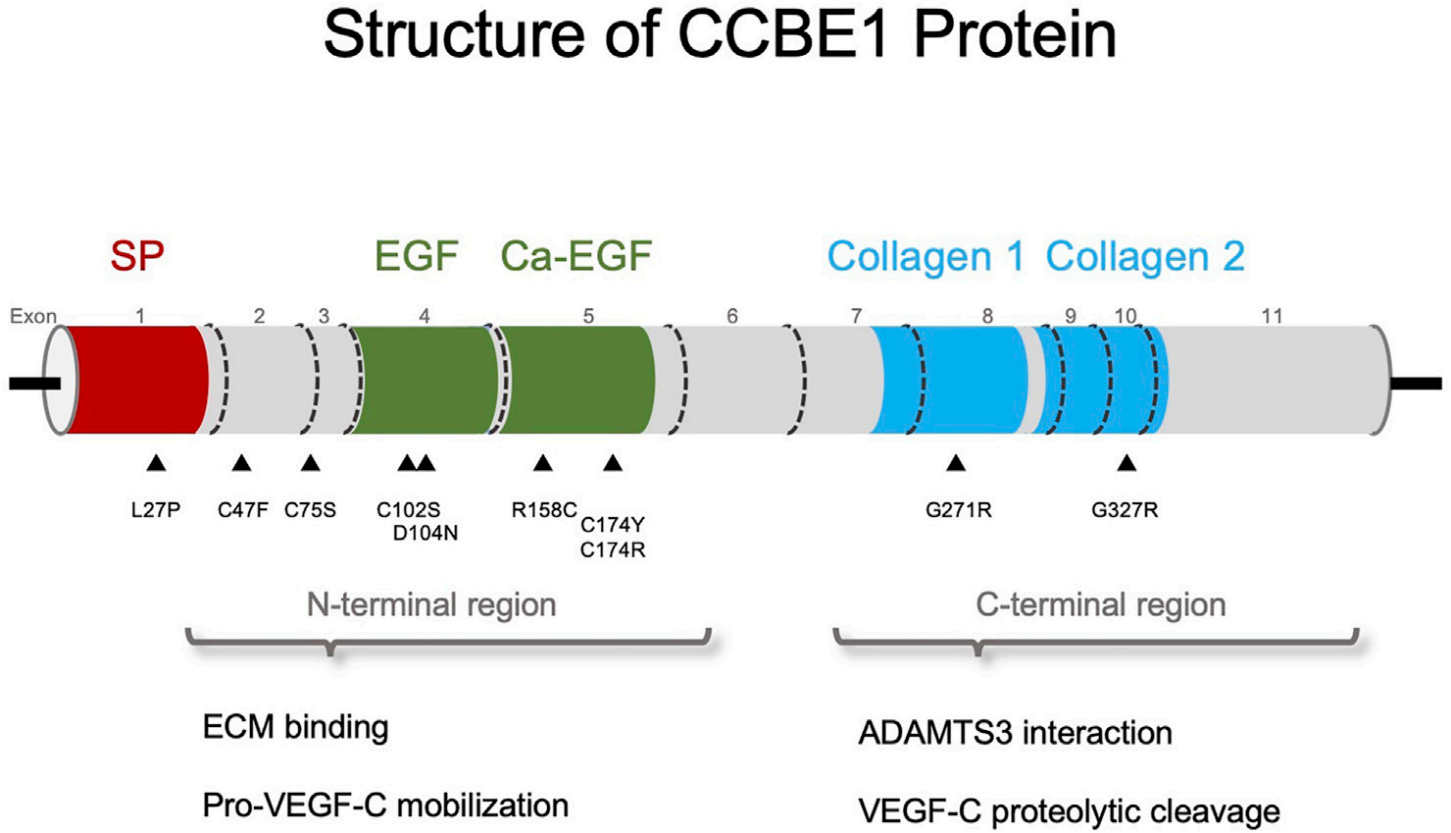
Schematic representation of CCBE1 domains: signal peptide (SP) in red, EGF and calcium-binding EGF (Ca-EGF) domains in green, and collagen repeats (Collagen 1, Collagen 2) in cyan. The triangles indicate the CCBE1 mutations screened in Hennekam syndrome patients. The N-terminal region is described to bind to the ECM and important for the mobilization of pro-VEGF-C. The C-terminal region of CCBE1 interacts with ADAMTS3 promoting the proteolytic cleavage of pro-VEGF-C.

The *CCBE1* gene encodes for a 408 amino acid (44 kDa) ECM protein containing a signal peptide for secretion, a calcium-binding epidermal growth factor (EGF)-like and EGF domains at the N-terminal, and at the C-terminal two collagen-like repeats (Figure 1) (Alders et al., 2009; Jeltsch et al., 2014). Moreover, at the C-terminal domain, CCBE1 displays post-translational modifications which include one chondroitin sulfate and two glycosylated sites (Figure 1) (Bui et al., 2016). CCBE1 is localized in secretory vesicles (Alders et al., 2009) due to exocytosis and is released into the ECM, where it binds to collagens or vitronectin proteins (Bos et al., 2011). Accordingly, Ccbe1 functions in a non-cell-autonomous manner in zebrafish (Hogan et al., 2009). Regarding its function, CCBE1 was first described as an essential molecule for lymphatic development in zebrafish and in mouse (Hogan et al., 2009; Bos et al., 2011; Hägerling et al., 2013; Le Guen et al., 2014). In humans, besides its association with HS (Hennekam et al., 1989; Van Balkom et al., 2002; Alders et al., 2009; Connell et al., 2010; Connell et al., 2012; Alders et al., 2013; Shah et al., 2013), CCBE1 has also been described as relevant protein in several types of cancer such as ovarian, breast and colorectal cancer, as well as gastrointestinal stromal tumor (GIST) and tumor lymphangiogenesis (Barton et al., 2010; Tian et al., 2016; Mesci et al., 2017; Zhao et al., 2018; Song et al., 2020).

The current knowledge about the action mechanism of CCBE1 has been mostly obtained in the context of lymphangiogenesis. Using a genetic screen in zebrafish, *ccbe1* was identified as indispensable for embryonic lymphangiogenesis (Hogan et al., 2009). Ccbe1 activity is described to be exerted at the same stage of development as the vascular endothelial growth factor-C (Vegf-c) and Vegf receptor 3 (Vegfr-3), being required in zebrafish for lymphangioblast budding and for angiogenic sprouting from venous endothelium (Hogan et al., 2009). When *Ccbe1* was ablated in mice (Bos et al., 2011), the phenotype was closely phenocopying the *Vegfc* knockout (Karkkainen et al., 2004). Bos et al. reported that CCBE1 is required for murine embryonic lymphangiogenesis but not for angiogenesis, independent of VEGFR-3 phosphorylation. In addition, this study showed that a recombinant version of CCBE1 leveraged a lymphatic response driven by VEGF-C in a corneal micropocket assay (Bos et al., 2011). Finally, *in vitro* experiments using Human Umbilical Vein Endothelial Cells (HUVEC) suggests that human CCBE1 protein probably exerts this effect by binding the N-terminal EGF-like domains to the ECM components collagen I, IV, V, and vitronectin (Bos et al., 2011). However, further analysis would be needed to confirm this finding *in vivo*. In conclusion, CCBE1 was recognized to be necessary for the VEGF-C/VEGFR-3 signaling pathway.

Interestingly, it was also demonstrated that when co-transfected with VEGF-C in Human embryonic kidney 293 (HEK293) cells, CCBE1 enhanced the processing of the full-length form of VEGF-C (Jeltsch et al., 2014). The collagen domains of CCBE1 interact with ADAMTS3 (A disintegrin and metalloproteinase with thrombospondin motifs-3) protease promoting proteolytical cleavage of VEGF-C pro-peptides into its active mature form (Jeltsch et al., 2014). Furthermore, *in vitro* assays using porcine aortic endothelial (PAE) cells demonstrated that the N-terminal domain of CCBE1 increases VEGF-C activity enhancing pro–VEGF-C cleavage to the mature form (Jeltsch et al., 2014). The same year, Le Guen and others demonstrated that the zebrafish embryo requires Ccbe1 for normal Vegf-c/Vegfr-3/Erk signaling (Le Guen et al., 2014). In addition, *in vitro* experiments showed that CCBE1 increased the levels of mature processed VEGF-C in *trans* and hence performed its function outside of the cell (Le Guen et al., 2014). Therefore, it seems that CCBE1 is able to increase levels of both partially processed and full-length forms of VEGF-C, suggesting that CCBE1 activates VEGF-C through its processing and release (Jeltsch et al., 2014; Le Guen et al., 2014). In concordance with these results, accumulation of the unprocessed form of VEGF-C was also found in the embryonic heart of *Ccbe1* mutant mice (Bonet et al., 2018).

Roukens et al., in an attempt to understand the functional role of the different CCBE1 protein domains, generated distinct knock-in mice harboring other *CCBE1* mutations deleting either the EGF and Ca-EGF domains (N-terminal) or both collagen-repeat domains (C-terminal). Lymphatic structures were completely absent in both *Ccbe1* null mice and those lacking the C-terminal domain of CCBE1. In contrast, in mice where CCBE1 N-terminal domain was depleted displayed some clusters of lymphatic endothelial cells (LECs; Roukens et al., 2015). These results, together with *in vivo* and *in vitro* assays performed in zebrafish and HEK293 cells, respectively, suggest that C-terminal (collagen domains) of CCBE1 is crucial for the activation of VEGF-C. In contrast, the N-terminal harboring the EGF domains seems to be redundant for regulating VEGF-C *in vitro* processing but necessary for the *in vivo* lymphangiogenic activity of CCBE1 (Roukens et al., 2015). Accordingly, co-transfection assays in HEK293 cells showed that a truncated form of CCBE1 lacking its collagen-like domain (C-terminal) were unable to carry out VEGF-C processing (Bui et al., 2016). While these data contradict prior studies (Bos et al., 2011; Jeltsch et al., 2014), the idea that CCBE1 N-terminal domain is crucial for the proper processing of pro-VEGF-C was supported by Jha et al. In this study, PAE cells stably expressing VEGFR-3 were exposed to pro-VEGF-C in the presence and absence of the N-terminal domain of CCBE1 (CCBE1-175). Notably, only the presence of CCBE1-175 led to a reduction in the amount of pro-VEGF-C protein (Jha et al., 2017). Accordingly, proteomic analysis revealed higher levels of mature VEGF-C in Ba/F3-hVEGFR-3/EpoR cells treated with recombinant CCBE1-175 than those treated with pro-VEGF-C alone or a mixture of pro-VEGF-C and CCBE1-CollD (CCBE1 C-terminal). Finally, this study demonstrated that only the CCBE1 N-terminal domain stimulates VEGF-mediated VEGFR-3 phosphorylation, thus promoting cell survival in VEGFR-3-expressing PAE (Jha et al., 2017).

One possible reason for the contradicting data on the domains of CCBE1 could be the low efficiency reported in the production of full-length recombinant CCBE1 in eukaryotic systems (Jeltsch et al., 2014; Roukens et al., 2015; Bui et al., 2016). In fact, it was observed a retention of recombinant CCBE1 proteins in the endoplasmic reticulum upon overexpression (Silva et al., 2018). This might be caused by a saturation of the folding machinery of the cell when CCBE1 is overexpressed, which forced Jeltsch et al. to produce the N-terminal domain variant CCBE1Δ175 on their experiments that might not full-copy CCBE1 plentiful function (Jeltsch et al., 2014). A strategy to increase the yield in full-length rCCBE1 production was more recently reported (Silva et al., 2018), thus providing a suitable tool for further validation of CCBE1 and its domains role *in vitro*. Interestingly, it is important to note that most of the mutations identified in HS patients affect the N-terminal domain of CCBE1, in contrast with the only two mutations found at the C-terminal (collagen domains) (Roukens et al., 2015). This observation is in consonance with the *in vitro* experiments that described a minor effect on CCBE1 function upon N-terminus (EGF and Ca-EGF domains) deletion than the collagen domains (C-terminal). However, until the development of a proper *in vivo* system, the role of the N- and C-terminal domain in the protein function will remain elusive.

On the other hand, it is considered that both CCBE1 domains are independently involved in VEGF-C processing and activation but using different mechanisms. While CCBE1 N-terminal domain immobilizes pro-VEGF-C facilitating the formation of the CCBE1/ADAMTS3/pro-VEGF-C complex, the C-terminal domain might act as a coenzyme accelerating the proteolytic cleavage of pro-VEGF-C generating the active protein (Jeltsch et al., 2014).

Finally, besides its well-characterized interaction with ADAMTS3, CCBE1 has also been indicated to interact with the protease kallikrein-related peptidase 3 (KLK3). KLK3, also known as the prostate-specific antigen (PSA), is a major protease found in human semen capable of cleaving VEGF-C at a novel N-terminus site and whose activity is enhanced in the presence of CCBE1 (Jha et al., 2019).

## Expression and Function During Early Heart Development

Although some HS patients show evidence of heart defects, the contribution of CCBE1 to the development of the heart only recently started being explored. In this context, *CCBE1* was first identified on a differential screen designed to identify genes expressed in vertebrate cardiac progenitors (Bento et al., 2011). In the chicken embryo, gene expression analysis confirmed the expression of *CCBE1* in the bilateral cardiogenic mesoderm, also known as heart forming regions, until later when these regions fuse at the midline at Hamburger Hamilton (HH)9 stage to form the primitive heart tube (Furtado et al., 2014). Expression of *CCBE1* in the heart-forming regions coincides with both the FHF and SHF progenitor regions. However, it is detected on either side of the primitive streak at HH4 even before the formation of the two heart field populations, but where the cells with cardiogenic potential are located (Furtado et al., 2014). Upon the formation of the heart tube, *CCBE1* expression was also detected close to the pharyngeal mesoderm where the SHF progenitors locate (Furtado et al., 2014). A study in mice also confirmed *Ccbe1* expression in both FHF and SHF heart progenitors (Facucho-Oliveira et al., 2011). Additionally, this study reported the expression of *Ccbe1* in the extracardiac progenitor tissue PE (Facucho-Oliveira et al., 2011). Later, from embryonic day E11 until E13.5, *Ccbe1* expression has been reported in the heart mesothelium, better known as pericardial wall (Bos et al., 2011; Facucho-Oliveira et al., 2011; Hägerling et al., 2013). In addition, *Ccbe1* transcripts have been detected in cardiac fibroblasts isolated from embryos around E13.5 (Ieda et al., 2009).

These findings indicating that *Ccbe1* is expressed in cardiac populations suggest that CCBE1 could play a role during early cardiogenesis. In this regard, the knockdown of *CCBE1* in the chicken embryo results in defective heart tube formation as the bilateral heart-forming regions could not fuse at the midline (Furtado et al., 2014). This could be explained by the defective migration of the cardiac precursor cells to the midline. In addition, *CCBE1* knockdown also causes a decreased proliferation of the cardiac precursor cells, suggesting that CCBE1 somehow might regulate cell proliferation. In contrast, the overexpression of *CCBE1* in the chicken embryo increased proliferation of cardiac precursor cells (Furtado et al., 2014). Furthermore, the bilateral forming regions of *CCBE1*-overexpressing chicken embryos do not migrate towards the midline, forming instead two bilateral and independent heart tubes defined as cardia bifida (Furtado et al., 2014). Since VEGF-C/VEGFR3 signaling has no reported functions in these contexts, it is tempting to speculate that CCBE1 has alternative functions in regulating migration and proliferation during avian heart development that is independent of ADAMTS3-mediated processing of VEGF-C.

The first published results on *Ccbe1* mutant mice reported no obvious heart defects (Bos et al., 2011; Zou et al., 2013; Jakus et al., 2014). Bos et al. described that *Ccbe1* mutant mice lacking exons 1 and 2 did not present heart defects, even though they presented lymphedema and died at E14.5 (*Ccbe1* ^*tm1Lex*^ mouse line; Bos et al., 2011). However, and in contrast with this study on the role of CCBE1 in lymphangiogenesis, the *Ccbe1* mutant strain lacking exon 3 survives until birth with no obvious edema at E18.5 despite the defective lymphatic vasculature (Zou et al., 2013; Jakus et al., 2014). This discrepancy might be explained by two facts. Firstly, the original *Ccbe1* mutants were generated in a pure C57Bl/6 genetic background (Bos et al., 2011), while the mice used in this study have a mixed 129SV/C57BL/6 background (Zou et al., 2013; Jakus et al., 2014). Differences in genetic background have been reported to impact the penetrance of the phenotype observed in fetal liver erythropoiesis in *Ccbe1* mutant embryos (Zou et al., 2013). Therefore, we must not exclude the existence of uncharacterized or transient genetic background-dependent cardiac defects in the *Ccbe1* mutants. Secondly, the original *Ccbe1* mutant line was generated by the removal of the exons 1-2 (Bos et al., 2011) whereas the second one by the removal of the internal exon 3 (Zou et al., 2013; Jakus et al., 2014). The fact that exons 1-2 encode for the signal peptide could result in a much more compromised form of the CCBE1 protein than the removal of exon 3, whose deletion neither cause any frameshift nor subtract any apparently essential domain.

In agreement with a possible role during cardiogenesis in mice, disruption of normal CCBE1 activity by shRNA knockdown (KD) or by a blocking antibody in differentiating mouse embryonic stem cells (mESCs) *in vitro,* results in a strong decrease in the expression of several cardiac lineage markers without affecting the pan-mesoderm marker BRACHYURY (Bover et al., 2018). Moreover, this *in vitro* model for cardiac differentiation demonstrated that, as occurs during mouse and chick cardiac development, high *Ccbe1* expression correlates with the onset of cardiac specification, as observed in SHF and PE cardiac progenitors (Facucho-Oliveira et al., 2011; Furtado et al., 2014; Bover et al., 2018). In addition, *Ccbe1* KD resulted in an impairment of embryoid body (EB) growth caused by reduced cell proliferation and a relative increase in cell death, especially from day 4 onwards (Bover et al., 2018). This cardiac fate during mESCs differentiation is EB size, growth factor signaling and ECM proteins niche dependent, but in particular, how mESCs interact with this developmental niche (Czyz and Wobus, 2001; Bratt-leal et al., 2009; Goh et al., 2013; Higuchi et al., 2013; Taylor-Weiner et al., 2013; Zeng et al., 2013). However, after blocking CCBE1 activity using anti-CCBE1 antibodies, the expression of *Mesp1* was also affected even before the difference in size could be detected, indicating that the normal cardiac mesoderm commitment is dependent on normal levels of CCBE1 and not a consequence of the reduced size of the EBs (Bover et al., 2018). This showed that CCBE1 is important and specific for the proper cardiac specification *in vitro*. More specifically, CCBE1 is necessary for the formation of the cardiogenic mesoderm (*Mesp1*), cardiac progenitors (*Isl1*), and the formation of mature cardiomyocytes (*αMhc*, *cTnt*). Hence, the reduced proliferation rate observed in the *in vitro* differentiation of *Ccbe1* KD mESCs seems to be crucial for cardiomyocyte formation.

## CCBE1: Role in Coronary Vasculature Development

Coronary vascular formation is a fundamental event in the developing heart that involves the formation of a primitive plexus of ECs that progressively expands into a vascular network that completely vascularizes the myocardium (Zeini et al., 2009; Red-Horse et al., 2010). The embryonic coronary endothelium arises from three different progenitor populations of ECs: the ventricular endocardium (Wu et al., 2012), the SV endocardium (Red-Horse et al., 2010; Chen et al., 2014a) and the PE (Katz et al., 2012; Cano et al., 2016). Lineage tracing experiments indicated that SV-derived coronary ECs mainly colonize the dorsal aspect and the right lateral side of the heart (Chen et al., 2014a), whereas the ventricular endocardium-derived cover the ventral side (Wu et al., 2012; Sharma et al., 2017). On the other hand, proepicardial endothelial progenitor cells populate evenly and intramyocardially the ventricular wall (Katz et al., 2012; Cano et al., 2016).

**FIGURE 2:**
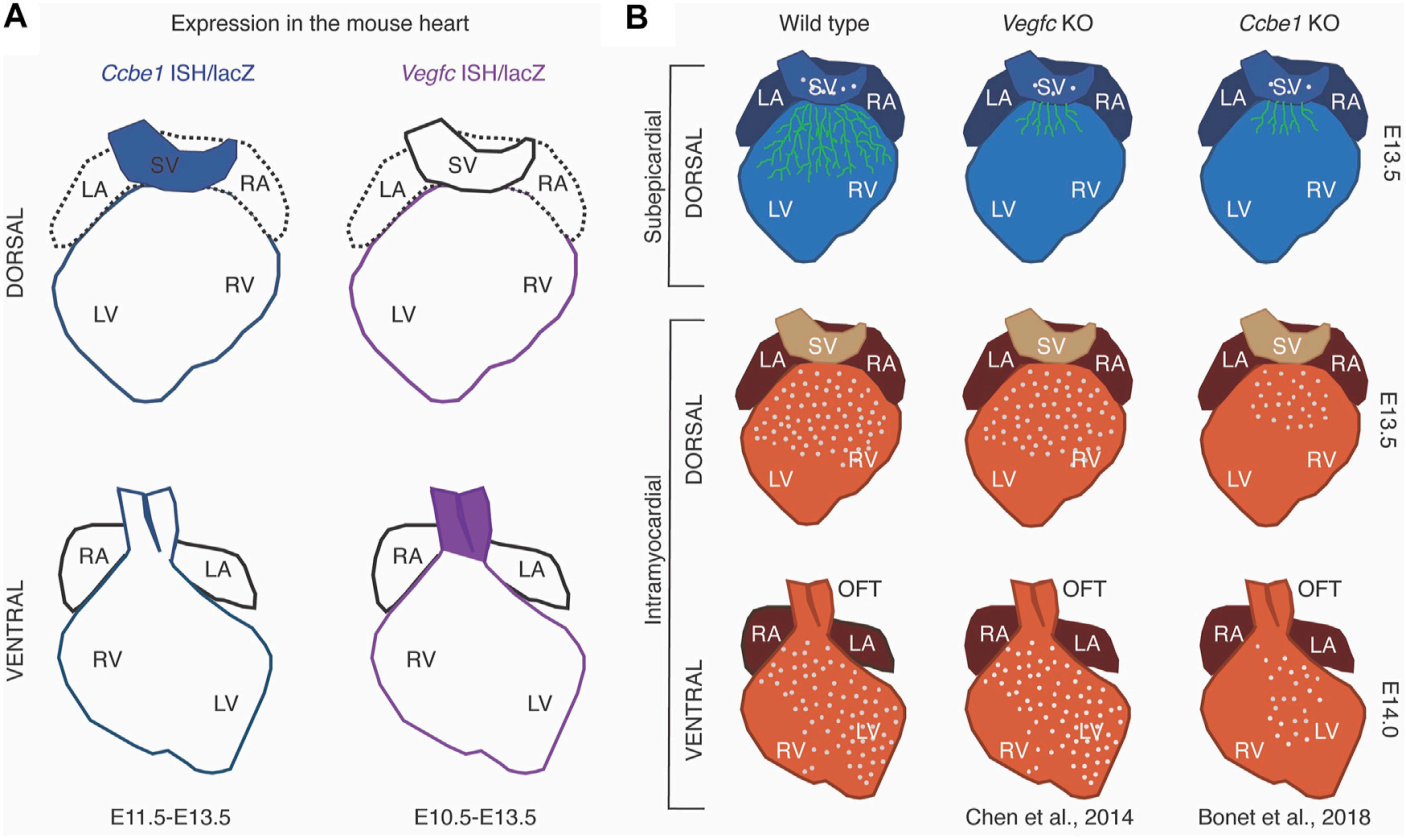
Schematic of *Ccbe1* and *Vegfc* expression and heart phenotypes. **(A)**: *Ccbe1* and *Vegfc* expressions are colocalized in the epicardium from E10.5 onward. *Ccbe1* expression is also present in the SV. *Vegfc* is expressed in the vessel wall of the aorta and pulmonary artery, whereas *Ccbe1* is restricted to the aortic epicardium. **(B)**: Schematic representation of heart phenotype in *Ccbe1* KO vs *Vegfc* KO mice shows that both mutant lines display underdeveloped dorsal subepicardial coronary vessels, however, Ccbe1 phenotype extends to defective dorsal and ventral intramyocardial vessels. Green draws represent subepicardial vessels. Gray dots represent intramyocardial vessels.

**FIGURE 3:**
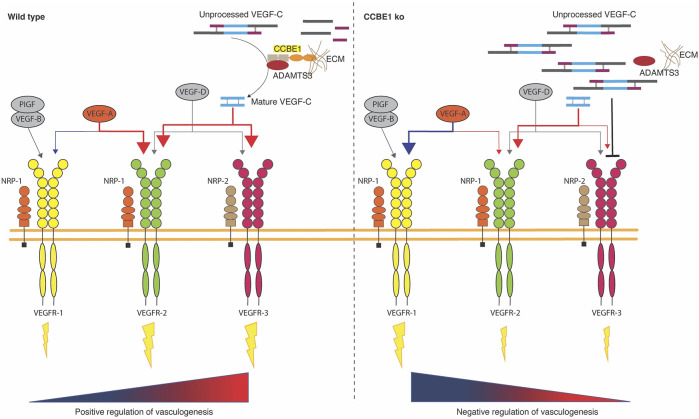
The VEGF family and receptor activation in the presence and absence of CCBE1. Left panel: CCBE1-ADAMTS3 complex promotes the processing of the mature form of VEGF-C, which positively regulates vasculogenesis *via* VEGFR-2 and -3 activation. Right panel: absence of CCBE1 leads to an accumulation of the unprocessed form of VEGF-C. Unprocessed VEGF-C binding to VEGFR-3 does not increase VEGFR-3 signaling but displaces mature VEGF-C from VEGFR-3. Displaced mature form of VEGF-C competes with VEGF-A for binding to VEGFR-2 and enhancing the binding-affinity of VEGFR-1 for VEGF-A, which translates in negatively regulation of vasculogenesis.

As mentioned previously, *mCcbe1* expression was detected in the PE of the mouse embryos at E9.5 and in the mesothelium of the parietal pericardium at E10.5 (Facucho-Oliveira et al., 2011). In another study, using the mouse line *Ccbe1* ^*tm1Lex*^, in which the coding exons 1 and 2 of the *Ccbe1* gene were replaced by the *lacZ* cassette, and X-Gal staining to detect β-galactosidase activity, *Ccbe1* expression was found in the pericardium and in the proximity of the cardinal veins of the mouse heart from E10.5 to E12.5 (Bos et al., 2011). The fact that cardinal veins merge on each side to form the sinus horns which enter the SV, places *Ccbe1* expression in the vicinity of the SV, however, no expression in coronary endothelium progenitors was detected (Bos et al., 2011). In contrast, a recent study reported that *Ccbe1* is expressed in the SV myocardium and in the epicardium at the stage in which coronary vessels start to form (E11.5-E13.5) (Bonet et al., 2018). Although both studies used the same approach to detect the expression of *Ccbe1* by examining β-galactosidase activity in *Ccbe1* ^*tm1Lex*^ heterozygous embryos (E11.5–E13.5), the discrepancy between results is likely due to the use of the Salmon-Gal staining in the second report (Bonet et al., 2018), a more sensitive method for ß-galactosidase detection than the traditional X-Gal staining (Sundararajan et al., 2012). In addition, the expression of *Ccbe1* in the epicardium and SV was confirmed by *in situ* hybridization method (Bonet et al., 2018). Accordingly, the subset of SV endocardium-derived dorsal subepicardial coronary vessels are severely underdeveloped in the *Ccbe1* ^*tm1Lex*^ mice lacking *Ccbe1*, suggesting that CCBE1 is necessary for the SV endocardium-derived subepicardial coronary vessels on the dorsal side of the heart (Bonet et al., 2018). In 2014, Chen et al. reported that VEGF-C is necessary for the proper development of SV endocardium-derived coronary vessels at the dorsal side of the heart, activating vessel migration along the surface of the ventricles (Chen et al., 2014a). This study shows that *Vegfc* is expressed in the epicardium (E10.5-E13.5) and dorsal subepicardial vessels are missing in *Vegfc* knockout (KO) hearts (Chen et al., 2014a). In this aspect, the similarity between phenotypes of *Ccbe1* and *Vegfc* mutant hearts is consistent with the CCBE1 requirement for the maturation of VEGF-C (Bos et al., 2011; Le Guen et al., 2014; Chen et al., 2014a; Jeltsch et al., 2014; Jha et al., 2017; Bonet et al., 2018). In concordance, the *Ccbe1* KO phenotype was accompanied by an accumulation of the unprocessed form of VEGF-C (Bonet et al., 2018), thus revealing that the role of CCBE1 in the processing of VEGF-C extends beyond the context of lymphangiogenesis. Surprisingly, *Ccbe1* mutant hearts display a broader defect that extends to dorsal and ventral intramyocardial coronary vessels (Figure 2) (Bonet et al., 2018), suggesting that CCBE1 is also required for the development of the coronary vessels derived from distinct embryonic cell sources, such as endocardial- and epicardial-derived. The discrepancy between phenotypes might be explained by the involvement of CCBE1 in distinct signaling pathways implicated in the development of ventral (endocardial-derived) coronary vessels such as VEGFR-2-dependent VEGF-A signaling (Wu et al., 2012). However, no signs of VEGF-A signaling pathway disruption were found in *Ccbe1* mutant hearts (Bonet et al., 2018). Nevertheless, CCBE1 could be involved in still unidentified signaling pathways required for the development of ventral coronary vessels. On the other hand, it has been demonstrated that the proteolytic activation of VEGF-C *via* CCBE1 enables the binding with high affinity to VEGFR-3 (Bui et al., 2016). In the adult, VEGFR-3 is restricted to LEC, however, during development, this receptor is present in all endothelia (Joukov et al., 1997). Apart from its well-characterized role in lymphatic vasculature development, VEGFR-3 has also been shown to be important for sprouting from pre-existing blood vessels, which need previous VEGFR-2 signaling to be responsive to VEGFR-3 ligands (Tammela et al., 2008). VEGFR-2 is a receptor expressed by most blood ECs that promotes endothelial proliferation and migration upon binding VEGF family (Chen et al., 2014a). The mature and active form of VEGF-C also binds VEGFR-2 (Joukov et al., 1997), which are particularly present in the SV (Chen et al., 2014a). Hence, CCBE1 might be relevant in VEGF-C signaling through VEGFR-2 for the formation of SV endocardium-derived coronary vessels (Chen et al., 2014a), since they are defective in *Ccbe1* KO mice (Bonet et al., 2018). Although the immature (∼58 kDa) form of VEGF-C can bind to VEGFR-3, the maximal receptor-stimulating activity occurs only upon binding of the 21 kDa mature VEGF-C protein (Joukov et al., 1997). In *Ccbe1* mutant mice, the immature/mature VEGF-C stoichiometry is altered due to the unprocessed ∼58 kDa form accumulation. In this regard, the mature form of VEGF-C (∼21 kDa) is detected in both wild type and *Ccbe1* mutant hearts (Bonet et al., 2018). This could affect the VEGFR-2 signaling as the accumulation of unprocessed form of VEGF-C might compete with its mature form for binding VEGFR-3, increasing therefore the affinity of VEGF-C for VEGFR-2. Since the binding of VEGF-A and -C to VEGFR-2 involves overlapping sites of the receptor (Joukov et al., 1997), this could displace VEGF-A from VEGFR-2, thus decreasing its availability for activation of VEGFR-2 and enhancing the binding-affinity of VEGFR-1, a negative regulator of angiogenesis during the development of the vascular system, for VEGF-A (Figure 3) (Fong et al., 1995; Fong et al., 1999). Accordingly, it has been shown that the binding of VEGF-C to VEGFR-3 may regulate VEGFR-2 signaling (Hamada et al., 2000). This effect might extend to SV- and endocardial-derived coronary vessels growth as both are capable of responding to VEGF-A and VEGF-C (Chen et al., 2014a).

During coronary vasculature development, coronary artery (CA) stems connect the primitive coronary plexus to the aorta to deliver oxygenated blood to the ventricular myocardium efficiently (Waldo et al., 1990). Significant human disease is associated with congenital anomalies in CA patterning (Angelini, 2007; Bashore, 2007). CCBE1 was also reported as an essential molecule for peritruncal vessel establishment and subsequent CA stem formation (Bonet et al., 2018). First, *Ccbe1* mutant mice display disrupted peritruncal vessel growth and the absence of subepicardial aortic vessels (Bonet et al., 2018). Second, CA stems are completely absent in E12.5 and E13.5 *Ccbe1* mutant hearts. Interestingly, 93% of hearts analyzed showed immature and abnormally low CA stems concerning the aortic root (E14.5). These results suggest that the mispatterned CA stems are consequence of a delay in the growth of peritruncal vessels that reach the aortic root at a later stage than in wild type animals. The fact that *Vegfc* mutant hearts display a similar phenotype (Chen et al., 2014b) supports that CCBE1 enhances the proteolytic processing of VEGF-C in the developing heart. Accordingly, *Ccbe1* expression was also found in the aortic epicardium. This study hypothesized that this CCBE1 secreted from the aortic epicardium into the subepicardial space is enough to efficiently process VEGF-C expressed throughout the outflow tract (Chen et al., 2014b) inducing therefore peritruncal vessel growth into the aortic wall.

## Transcriptional Regulation of CCBE1

Little is known about the mechanisms by which *CCBE1* is regulated at the transcriptional level. While only a few studies have addressed this point in the context of lymphangiogenesis and cancer, to date, nothing is known about the mechanisms that transcriptionally regulate *CCBE1* in the heart.

The E2F family of transcription factors plays a crucial role in the regulation of cell cycle progression (Dyson, 1998; Helin, 1998; Wenzel et al., 2011). In 2013, *VEGFR-3* and *CCBE1* were reported to be directly regulated by the atypical E2fs, E2f7 and E2f8 (E2f7/8), playing therefore an essential role in lymphangiogenesis (Weijts et al., 2013). Loss-of-function of E2f7/8 in zebrafish impairs lymphangiogenesis and venous sprouting through transcriptional regulation of *ccbe1* and *flt4* (Weijts et al., 2013). The role of E2F family during heart development lingers unstudied and no clues point to a role regulating *ccbe1* expression in the heart. However, E2F family has been described as a regulator of EC proliferation during cardiac neovascularization in a mouse model of myocardial infarction (Zhou et al., 2013), and also to promote angiogenesis through transcriptional activation of *Vegf-a* (Weijts et al., 2012), indicating a specific role of E2F family in the regulation of vascular growth.

In the context of cancer, two studies performed in rectal cancer (RC) suggest that the gene related to the growth of tumor Stomatin-like protein 2 (*SLP-2*) might be regulating *CCBE1* in the genesis of lymphatic tubes (Zhang and Liu, 2017; Guo et al., 2018). First, both *CCBE1* and *SLP-2* were associated with the prognosis of RC (Zhang and Liu, 2017). Then, higher levels of *CCBE1* (mRNA and protein) were observed in lymphatic tubes from RC tissue than in those from adjacent tissue (Guo et al., 2018), confirming the critical role of CCBE1 in the genesis of the lymphatic tube of RC. Moreover, both mRNA and protein expression analysis revealed a positive correlation between *SLP-2* and *CCBE1* in RC tissues, consistent with the previous study. Finally, it was shown that downregulation of *SLP-2* expression suppresses *CCBE1* expression in an *in vitro* model of human LECs (Guo et al., 2018).

Most recently, TGF-ß signaling was reported to negatively regulate *CCBE1* during colorectal cancer (CRC) tumor lymphangiogenesis (Song et al., 2020). This study demonstrated that TGF-β suppresses the expression and lymphangiogenic function of *CCBE1* in cancer-associated fibroblasts and CRC cells. In addition, ChIP-qPCR assays showed that the downstream effectors of TGF-β Smad2/3 were recruited to the enhancer regions 1, 2, 3, and 4 of the *CCBE1* promotor region after TGF-β treatment in SW837 cells (Song et al., 2020). However, further analysis by luciferase assays confirmed that only the enhancer regions 3 and 4 are the functional binding regions of SMAD2/3 to the *CCBE1* enhancer (Song et al., 2020).

Several studies have also described a post-transcriptional regulation of *CCBE1* gene expression by microRNAs targeting its 3′UTR region. In this context, miR-330-3p was reported to target *CCBE1* in an *in vitro* model of breast cancer increasing the invasive capacity of this cell line (Mesci et al., 2017). On the other hand, a recent study observed an inverse correlation between miR-942-5p expression and *CCBE1* expression in CRC and that inhibition of *CCBE1* using si-*CCBE1* reversed the effects induced by miR-942-5p overexpression into CRC cells. Finally, this study demonstrated by luciferase assays that *CCBE1* is a direct target of miR-942-5p (Zhou et al., 2021).

## Future Perspectives

Heart disease is still one of the significant causes of mortality in our society, and its increase is expected in the following years. Regenerative medicine represents a hopeful way to repair the damaged heart tissue. Timely revascularization after myocardial infarction improves cardiac function and is key to preventing post-infarction pathophysiological remodeling. Despite the advance of surgical and catheter-based revascularization, there are patients who are either ineligible or demonstrate suboptimal responses to these therapies exposing the need to think of alternative treatment approaches. Therapeutic angiogenesis aims to trigger the growth of new blood vessels from pre-existing ones in order to re-supply blood flow. Either to nurture the endogenous production of new cardiomyocytes or the engineered myocardial tissues transplanted into the infarcted site, restoring blood supply in the hypoxic regions of the diseased heart will improve cell survival. Since rCCBE1 enhances vessel formation *in vitro* (Tian et al., 2016; Silva et al., 2018), we are convinced that CCBE1 biological activity might also increase neoangiogenesis cell-based therapies. Moreover, this secreted molecule seems to have significant cardiogenic potential prompting the production of multipotent cardiovascular progenitors necessary for myocardium regeneration, leading to an improvement of the cardiac function post-myocardial infarction. Nevertheless, more studies are still needed to understand the significance of CCBE1 *in vivo* better and delineate suitable strategies for improving therapies in ischemic heart disease: should CCBE1-expressing cells be injected into the infarcted heart or a combination of cells with controlled released rCCBE1 protein more efficient? The effectiveness of direct implantation of cells into the diseased tissue is still a challenge, but the delivery and retention of small molecules loaded on hydrogels have been bringing out promising results. Therefore, we anticipate that the administration of a rCCBE1 protein or smaller peptides mimicking its functional domains may be capable of inducing neo-angiogenesis and/or cardiac remodeling, eliciting its therapeutical test in human patients in the near future.

## Abbreviations

ADAMTS3, A disintegrin and metalloproteinase with thrombospondin motifs-3; CCBE1, collagen- and calcium-binding EGF-like domains 1; SV, sinus venosus; HEK293, Human embryonic kidney 293 cells; VEGF, vascular endothelial growth factor; VEGFR, vascular endothelial growth factor receptor; FHF, first heart field; SHF, second heart field; CNC, cardiac neural crest; EC, endothelial cell; ECM, extracellular matrix; HH, Hamburger Hamilton; HS, Hennekam syndrome; EGF, epidermal growth factor; LEC, lymphatic endothelial cell; PAE, porcine aortic endothelial; ESC, embryonic stem cell; mESCs, mouse embryonic stem cells; KD, knockdown; KO, knockout; EB, embryoid body; CA, coronary artery; SLP-2, Stomatin-like protein 2; CRC, colorectal cancer; Ca-EGF, calcium-binding EGF, SP, signal peptide.
